# Type II Collagen-Conjugated Mesenchymal Stem Cells Micromass for Articular Tissue Targeting

**DOI:** 10.3390/biomedicines9080880

**Published:** 2021-07-23

**Authors:** Shamsul Bin Sulaiman, Shiplu Roy Chowdhury, Mohd Fauzi Bin Mh Busra, Rizal Bin Abdul Rani, Nor Hamdan Bin Mohamad Yahaya, Yasuhiko Tabata, Yosuke Hiraoka, Ruszymah Binti Haji Idrus, Ng Min Hwei

**Affiliations:** 1 Tissue Engineering Centre, Faculty of Medicine, Universiti Kebangsaan Malaysia, Clinical Block, Jalan Yaacob Latiff, Cheras, Kuala Lumpur 56000, Malaysia; sshamsul@ppukm.ukm.edu.my (S.B.S.); shiplu56@gmail.com (S.R.C.); fauzibusra@ukm.edu.my (M.F.B.M.B.); ruszyidrus@gmail.com (R.B.H.I.); 2 Department of Orthopedic & Traumatology, Faculty of Medicine, Universiti Kebangsaan Malaysia, Clinical Block, Jalan Yaacob Latiff, Cheras, Kuala Lumpur 56000, Malaysia; rizal@ppukm.ukm.edu.my (R.B.A.R.); nhmycj@gmail.com (N.H.B.M.Y.); 3 Department of Biomaterials, Institute for Frontier Medical Sciences, Kyoto University, 53 Kawara-cho Shogoin, Sakyo-ku, Kyoto 606-8507, Japan; yasuhiko@frontier.kyoto-u.ac.jp; 4 Biomaterial Group, R&D Center, Nitta Gelatin Inc., 2-22, Futamata, Yao City, Osaka 581-0024, Japan; yo-hiraoka@nitta-gelatin.co.jp

**Keywords:** gelatin microsphere, MSCs micromass, antibody conjugation, protein G, cell homing

## Abstract

The tissue engineering approach in osteoarthritic cell therapy often requires the delivery of a substantially high cell number due to the low engraftment efficiency as a result of low affinity binding of implanted cells to the targeted tissue. A modification towards the cell membrane that provides specific epitope for antibody binding to a target tissue may be a plausible solution to increase engraftment. In this study, we intercalated palmitated protein G (PPG) with mesenchymal stem cells (MSCs) and antibody, and evaluated their effects on the properties of MSCs either in monolayer state or in a 3D culture state (gelatin microsphere, GM). Bone marrow MSCs were intercalated with PPG (PPG-MSCs), followed by coating with type II collagen antibody (PPG-MSC-Ab). The effect of PPG and antibody conjugation on the MSC proliferation and multilineage differentiation capabilities both in monolayer and GM cultures was evaluated. PPG did not affect MSC proliferation and differentiation either in monolayer or 3D culture. The PPG-MSCs were successfully conjugated with the type II collagen antibody. Both PPG-MSCs with and without antibody conjugation did not alter MSC proliferation, stemness, and the collagen, aggrecan, and sGAG expression profiles. Assessment of the osteochondral defect explant revealed that the PPG-MSC-Ab micromass was able to attach within 48 h onto the osteochondral surface. Antibody-conjugated MSCs in GM culture is a potential method for targeted delivery of MSCs in future therapy of cartilage defects and osteoarthritis.

## 1. Introduction

Osteoarthritis (OA) is a chronic degenerative disease of the joints characterized by articular cartilage degeneration of the knee, hip, or hand; synovitis; and the loss of extracellular matrix, accompanied by progressive pain and functional impairment [1]. OA is one of the leading causes of chronic pain and disability, with 17.1 million people living with disability globally [2]. Treatment of OA depends on its severity, and varies from conservative treatment to invasive surgical intervention such as joint replacement [3]. However, due to the risk of failure and morbidity in the conventional treatments, the potential applicability of cell therapy and tissue engineering for treating OA were explored [4].

Cell-based therapies for the treatment of OA are not foreign; numerous studies have reported beneficial effects [5,6]. The therapies feature delivery of cells, in particular, mesenchymal stem cells (MSCs) to the knee by means of direct injection or implantation [7,8]. The desired effects of MSCs therapy in the treatment of OA knees were mostly for its immunomodulatory properties in which it was known that MSCs-based therapies were approved to be used in graft-vs-host disease for immunosuppression [9]. Generally, cell therapies for OA are safe, albeit their reported efficacy from several randomized trials is quite controversial [10]. This is due to the high variability in the cell preparation and lack of rigors in the trials that preceded its clinical shortcoming [11]. An argument was made, in which the improvements seen among patients in the trials who received MSCs injection were only transient, only to be followed with significant cell apoptosis following injection [12].

The inclination towards cell-based therapies for OA, nonetheless, has led to continued research to tackle major drawbacks. It is known that the MSCs did not survive for long following intra-articular injections [12,13]. Previous reports have shown that MSCs can only survive within a short period of time (within 1–2 weeks) post-injection in addition to the lack of MSCs engraftment to the affected tissue [14,15]. Im et al. in their review recommended that, to solve this, an environment for MSCs to coalesce together in suspension is needed as the communication between cells is paramount for cell survival [12]. Furthermore, the issue with MSCs dispersal and low affinity binding towards the targeted tissue following injection should also be investigated.

With this in mind, cell apoptosis and tissue engraftment could be addressed by the fine tuning of the current tissue engineering approach. The observed cell apoptosis following intra-articular injection is expected due to several reasons; first, the inhabitable inflammatory environment of an OA knee, and second, the inability of MSCs to survive single-handedly in a suspension [12]. The MSCs however, managed to exert their anti-inflammatory paracrine effects prior to death [12,13]. A provision of non-invasive surface matrix or scaffold for MSCs to attach and grow could prolong their survival and prevent dispersion inside the knee joint, in which the utilization of injectable scaffolds using microspheres may serve as a conceivable solution.

Microspheres are historically known for their role in drug delivery systems and in vitro cellular expansion [11,16]. Little was known about their role in cell delivery until several reports demonstrated their safety and efficacy in delivering cells for cartilage regeneration in vivo [17,18]. The microsphere is the contact point for the cellular attachment and growth that ensures cell viability while providing a foundation for native chondrocytes to invade and regenerate [19]. Furthermore, a recent study reported that microspheres (specifically gelatin microspheres (GM)) increased the chondrogenic differentiation of mesenchymal stem cells (MSCs) [20]. As microspheres are of miniscule size, microsphere delivery does not require open surgery, as they easily fit inside the needle for intra-articular injection in humans [21]. However, the possibility of the injected microspheres–cells attaching and integrating with the affected tissue following injection remains a significant concern, as this is the first crucial step in cell or tissue regeneration.

Several techniques were developed to improve the homing of cells towards the damaged tissue [22]. These strategies included cell surface modifications that involved: (1) regulating the expression of cell surface receptors, (2) modifying native cell receptors, (3) conjugating new molecules not initially present on the cell surface, or (4) conjugating antibodies to the cell surface.

Previous works have managed to utilize the glycoengineering technique to conjugate antibodies to the cell surface for adherence to the tissue [23,24]. In this case, the palmitate conjugate of protein G acts as the mediator that connects the cell with the antibody.

Therefore, this study was aimed at determining the effect of palmitated protein G (PPG) intercalation and antibody conjugation on MSCs. Here, protein G was first palmitated (producing PPG), followed by intercalation into the MSCs membrane (PPG-MSCs). The second step involved conjugating type II collagen antibody to the PPG-MSCs. Type II collagen is commonly found in the extracellular matrix of cartilage; hence the antibody conjugation would provide high affinity binding of MSCs to the articular defects. The proliferation and differentiation potentials of both PPG-MSCs and antibody-conjugated PPG-MSCs (PPG-MSCs-Ab) in the monolayer and 3D states (GM) were tested. Finally, a preliminary proof of concept of the efficacy of targeted delivery of the PPG-MSC-Ab micromass to an osteochondral defect explant was tested in vitro.

## 2. Materials and Methods

### 2.1. Cell Culture and Immunophenotype Analysis

Ethics approval for this study was granted by Universiti Kebangsaan Malaysia Research Ethics Committee with the approval code UKM PPI/111/8/JEP-2018-458 on 11 October 2018. Bone marrow samples were collected as aspirates from patients undergoing total knee replacement procedures in Hospital Canselor Tunku Muhriz. All patients were informed with a patient information sheet and filled a consent form prior to tissue collection.

Five–ten mL of bone marrow aspirates (*n* = 6) were harvested, isolated and cultured in monolayer culture using F12: DMEM (1:1) supplemented with 10% fetal bovine serum (FBS; Biowest, Riverside, MO, USA), 1% antibiotic-antimycotic (Gibco, Grand Island, NY, USA), 1% glutamax (Gibco, Grand Island, NY, USA), and 1% vitamin C (Sigma-Aldrich, St. Louis, MO, USA) (FD). The cells were incubated at 37 °C in a humidified atmosphere containing 5% CO_2_. When the cells began to reach the near confluence stage, they were trypsinized with 0.25% trypsin/0.1% EDTA (Gibco, Grand Island, NY, USA) and passaged in 75 cm culture flasks at low seeding density. Cell cultures from each patient were maintained separately until further usage. For MSCs characterization analysis, the cells were tested at passage 1 until 3 by flow cytometry for surface marker expression to evaluate the stem cells properties according to the International Society of Cellular Therapy (ISCT) guidelines [25]. The cells were harvested with 0.05% trypsin EDTA, washed with 0.2% bovine serum albumin (BSA) in PBS, and stained with mouse anti-human CD29, CD44, CD45, CD73, CD90 anti-HLA-DR (BD Pharmingen, San Jose, CA, USA) and CD13 antibodies (Life Technology, Carlsbad, CA, USA). In brief, 2 × 10^5^ cells were suspended in 100 μL of 0.2% BSA in PBS and stained with individual antibodies at a concentration recommended by the manufacturer in separate tubes for 30 min. The cells were then washed with 0.2% BSA/PBS twice and fixed in 4% paraformaldehyde. Samples were washed twice in PBS, suspended in 0.2% BSA/PBS, and analyzed by FACS Calibur cytometer (BD Biosciences, San Jose, CA, USA) using Cell Quest Pro software. Ten thousand gated events were recorded. Gating was determined based on unstained controls.

### 2.2. Fabrication of Gelatin Microsphere

The gelatin microspheres (GM) were fabricated according to an established method [26]. Briefly, 4 g of gelatin was dissolved in 20 mL of water and heated up to 60 °C. Two hundred milliliters of olive oil were heated up to 40 °C. Gelatin was then added drop-wise into the olive oil while stirring at 420 rpm with a mechanical stirrer. The water-in-oil (*w/o*) emulsion was stirred for 10 min before being immersed into an ice bath to maintain the temperature at 10 °C and stirred for a further 30 min. Sixty milliliters of chilled acetone was then added, and the mixture was stirred for another 1 h. The gelatin microspheres were extracted from the olive oil by centrifuging and washing with chilled acetone. The non-crosslinked and dried GM was treated in a vacuum oven at 140 °C and 0.1 torr for dehydrothermal crosslinking of gelatin according to the method previously reported [27]. GM was characterized for their sizes using an optical microscope and their surface morphology using a scanning electron microscope (SEM). Images were taken using a digital camera attached to the optical microscope, and the sizes were analyzed by measuring the diameters of the microspheres using image QImaging Q-Capture Pro 7 (Surrey, BC, Canada). For imaging under SEM, dry microspheres were mounted onto aluminum stub and sputter-coated with Au/Pd and viewed under a scanning electron microscope in the high-pressure mode of 15 kV accelerating voltage.

### 2.3. Cell Coating

Recombinant protein G (Sigma, St. Louis, MO, USA) was derivatized with N-hydroxysuccinimide ester of palmitic acid (Sigma, St. Louis, MO, USA), as previously described [28]. The lipid-derivatized protein G was purified on a 10 mL Sephadex G-25 (Pharmacia, Piscataway, NJ, USA) column equilibrated with PBS containing 0.1% deoxycholate (DOC) pH-7.4. The protein concentration was adjusted to 750 µg/mL by OD absorbance at 280 nm, 20 µm filter sterilized, and stored at 4 °C until use. Briefly, in vitro expanded MSCs were incubated in 0.25% trypsin/0.1% EDTA (Sigma-Aldrich) for 5 min at 37 °C, collected, washed three times in serum-free DMEM, and resuspended at a density of 2 × 10^6^/mL in DMEM. Varying concentrations of palmitated protein G (PPG) or non-derivatized protein G (as a negative control) were added to the cell suspension, and the mixture was incubated at 37 °C for 2 h with constant gentle mixing. The PPG coated MSCs (PPG-MSCs) were washed in 2 mL of Hank’s balanced salt solution (HBSS) three times, centrifuging at 400× *g* for 5 min between each wash. The PPG-MSCs were then incubated in targeting antibody 100 µg/mL per antibody in PBS for 1 h at 4 °C. The targeting antibodies were antibodies to type II collagen (DSHB Cat:II-II6B3, RRID:Ab 528165, Iowa City, IA, USA). To assess the incorporation of PPG onto MSCs surfaces, cells incubated in different concentrations of PPG in PBS plus 0.1% DOC or cells incubated in buffer alone for 2 h were washed twice in the buffer and then incubated at 4 °C for 1h with 100 µL (per 1 × 10^6^ cells) of 100 µg/mL of FITC-human IgG (Sigma, Cat: F9512) diluted in PBS plus 0.1% DOC. PPG-MSCs were washed three times in the buffer and analyzed by flow cytometry and Nikon Eclipse Ti fluorescence microscope (Nikon, Tokyo, Japan).

### 2.4. Preparation of Cell Differentiation and Characterization

GM was sterilized with 70% ethanol, followed by complete washing with sterilized phosphate buffer saline (PBS; Sigma-Aldrich). PVA (Sigma-Aldrich) with a polymerization degree of 1800 and percent saponification of 88 mole % was dissolved in PBS. This solution (1 mL/well) was added into each well of 12- and 24-well and incubated at 37 °C for 15 min. The solution was then removed by aspiration and the well washed with PBS (1 mL/well) twice. For differentiation experiments, the microspheres were transferred to 12-well plates at 10 mg per well, and 5 × 10^4^ PPG-MSCs were seeded onto the microspheres per well (i.e., 5 × 10^3^ cells per mg of microspheres). For cell proliferation experiments, the microspheres were transferred to 24-well plates at 2 mg per well, and 1 × 10^4^ PPG-MSCs per well were subsequently seeded onto the microsphere. Presto Blue assay (Thermo Fisher Scientific, Waltham, MA, USA) was applied to study the proliferation of PPG-MSCs on monolayer culture (at day 3 and 5) and GM (at day 1, 3 and 7). All the respective culture and blank plates were rinsed with PBS, and then 180 µL of FBS-free medium was added into each plate, followed by 20 µL of Presto Blue reagent on top of the FBS-free medium. Later, the plates were incubated for 2 h at 37 °C and 5% CO_2_. After 2 h of incubation, 100 µL of the supernatant was transferred into a 96-well plate. Subsequently, fluorescence reading was measured using a microplate reader at an excitation wavelength of 570 nm and an emission wavelength of 600 nm.

### 2.5. Evaluation of MSCs Differentiation

The PPG-MSCs on monolayer and GM were cultured in three different culture media for multilineage differentiation as previously described [20,28,29]. For osteogenic differentiation, MSCs were cultured in FD medium (Gibco, Grand Island, NY, USA) supplemented with 0.1 mM dexamethasone (Sigma–Aldrich, St. Louis, MO, USA), 10 mM b-glycerol phosphate (Sigma–Aldrich, St. Louis, MO, USA), and 0.2 mM ascorbic acid-2-phosphate (Sigma–Aldrich, St. Louis, MO, USA). Induced cells were then replenished with fresh medium every 3 days, and the induction period lasted for 21 days. Differentiation activity was assessed with Alizarin Red staining, which was positively stained for calcium deposition. Briefly, samples were fixed with cold ethanol for 1 h, rinsed with PBS, and stained with Alizarin Red for 1 h. The excess stain was washed off using PBS, followed by incubation with boric acid buffer and counterstained with hematoxylin. Samples were dried and evaluated using a bright field microscope (Olympus-CK40, Tokyo, Japan). For quantification purposes, Alizarin Red dye was quantified using a solution of 20% methanol and 10% acetic acid in water. After 15 min, the liquid was transferred to a 96-well plate and read on the spectrophotometer at a wavelength of 450 nm.

In chondrogenic differentiation, PPG-MSCs was cultured in FD medium (Gibco, USA) supplemented with serum, 1% Insulin Transferring Selenium (ITS) (Gibco, Grand Island, NY, USA), 0.2 mM ascorbic acid-2 phosphate (Sigma, USA), 40 ng/mL L-proline (Sigma, St. Louis, MO, USA), 100 nM dexamethasone (Sigma–Aldrich, St. Louis, MO, USA), 10 ng/mL transforming growth factor-beta 3 (TGF-β3) (Invitrogen Inc., Waltham, MA, USA), and 50 ng/mL insulin-like growth factor 1 (IGF-1) (Invitrogen Inc., Waltham, MA, USA) [28,30]. The medium was changed every 3 days, and the induction period lasted for 21 days. The resulting culture was fixed and stained with toluidine blue to identify the presence of proteoglycans using a bright field microscope. Toluidine Blue dye was quantified using a solution made up of 2.5 mL of concentrated sulfuric acid and 2.5 mL of water in 95 mL of methanol. The liquid was transferred to a 96-well plate on a spectrophotometer and measured under a wavelength of 635 nm.

In adipogenic differentiation, MSCs were cultured in FD medium (Gibco, Grand Island, NY, USA) supplemented with 0.25 mmol/L 3-isobutyl-1-methylxanthine (Sigma–Aldrich, St. Louis, MO, USA), 100 nmol/mL dexamethasone, and 100 nmol/L human recombinant insulin (Sigma–Aldrich, St. Louis, MO, USA). The culture medium was changed every 3 days. After day 21, samples were stained with Oil Red O to identify lipid deposition. Briefly, adipogenic cultures were rinsed once with PBS and fixed with 10% formalin for 60 min at room temperature. Formalin was discarded, and the cells were stained with 0.36% Oil Red O for 50 min. The cells were subsequently examined using a bright field microscope. Oil Red O dye was quantified by adding 100% isopropanol, and incubated for 10 min. The liquid was transferred to a 96-well plate on a spectrophotometer and measured under a wavelength of 500 nm.

### 2.6. Immunofluorescence Staining

The samples were washed with DPBS and fixed with 4% paraformaldehyde overnight, permeabilized for 20 min with 0.5% Triton X-100 solution (Sigma-Aldrich), and then blocked with 10% goat serum for 1 h at 37 °C. The cells were incubated with Mouse Anti-Human Collagen II (DSHB Cat:II-II6B3, RRID:Ab 528165) for chondrocytes overnight at 4 °C. The following day, the cells were incubated with Alexa Fluor 594 goat anti-mouse antibodies (Invitrogen) for 1 h at 37 °C and counterstained with DAPI (Dako, Glostrup, Denmark) for 15 min. The cells were observed and evaluated with z-stack acquisition using a Nikon Eclipse Ti fluorescence microscope (Nikon, Japan).

### 2.7. Measurement of Real-Time PCR

Total RNA from MSCs, PPG-MSCs, GM-MSCs and GM-PPG-MSCs cultures was extracted using the Qiagen RNeasy mini kit. Concentrations of the extracted total RNA were determined using Nanodrop 2000 spectrophotometer (Thermo Fisher Scientific, Waltham, MA, USA). Complementary DNA (cDNA) was synthesized from the extracted RNA using the Maxima first strand cDNA synthesis kit (Thermo Scientific). The obtained cDNA was used for qPCR, which was performed in triplicates using the SYBR FAST Biorad qPCR master mix (Bio-rad, Hercules, CA, USA). The primers used in the qPCR are given in Table 1. Each sample was run in triplicates. For relative fold gene expression quantitation, the delta delta CT method was used. The CT value was calculated when the fluorescence of the sample exceeded the threshold level. Firstly, the average CT values across the triplicates were obtained for each sample. Then, the delta CT value was generated by normalizing the CT value of the gene of interest with the CT value of GAPDH. Finally, the relative fold change of the mRNA level of the target gene of each sample against the control was calculated as follows:

Relative fold change = 2^−∆∆Ct^

where, ΔCT = (CT target gene − CT GAPDH)

∆∆Ct = ∆Ct (sample) − ∆Ct (control)

### 2.8. Sulfated Glycosaminoglycan (sGAG) Production Assay

All samples were digested with a papain digestion solution (125 μg/mL papain, 5 mM L-cystein, 100 mM Na_2_HPO_4_ and 5 mM EDTA; pH 6.8) at 60 °C for 16 h. Sulfated glycosaminoglycan content was analyzed using a 1,9-dimethyl methylene blue (DMMB) assay (Biocolor, Belfast, UK). A 20 μL aliquot of each sample was pipetted into the microplate reader and added with 200 μL DMMB. Samples were analyzed immediately by measuring the absorbance at 525 nm [20].

### 2.9. Preliminary Assessment on Articular Cartilage Surface Binding

Articular cartilage tissues were collected from total knee replacement surgery of the consented patient. Cartilage tissues were excised from the distal femoral condyles. The harvested specimens were cleaned and they were carved into osteochondral explant (OC explant) using a 5-mm biopsy punch. The height of OC explant was around 1 cm. The OC explant were either cultured directly as an explant culture model for subsequent detailed characterization, or else they were processed for the explant co-culture model, using MSCs alone or GM-PPG-MSC-Ab for validation. To perform the explant co-culture, a focal defect of 2.5-mm diameter was first created at the center of the OC explant using a 2.5-mm biopsy punch. After the punch biopsy was created on each explant, the MSCs or GM-PPG-MSC-Ab, both pre-stained with CellTracker Green CMFDA (Molecular Probes Inc, Eugene, OR, USA), were injected to the biopsy site facing upward and let incubated at 37 °C. The explants were then flipped 180 °C inside the well for the unattached remnants to fall by gravity. After 48 h, the evidence of cell engraftment was assessed via fluorescence microscopy.

### 2.10. Statistical Analysis

Results were expressed as mean ± standard error of the mean (SEM) and analyzed using two-way ANOVA and Tukey’s multiple comparison tests. The differences were considered significant when *p* < 0.05.

## 3. Results

### 3.1. Stem Cell Characterization

**Figure 1:**
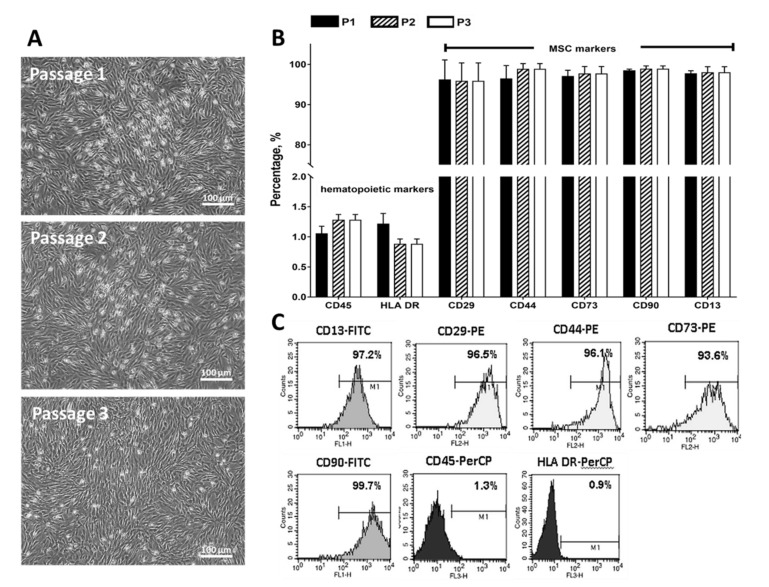
Characterization of Bone-Marrow Stem cells. (**A**) Morphology of MSCs in 7 days of culture at different passages under the light microscopy. (**B**,**C**) Percentage and flow cytometric analysis of MSCs surface markers were tested at passage 1 according to the ISCT guidelines. Scale bars indicate 100 µm.

MSCs appeared spindle shaped in all passages (1 to 3) (Figure 1A). All samples reached confluency within a week. Immunophenotyping of MSCs at passage 1, 2 and 3 was performed using a panel of markers based on the International Society for Cell Therapy guidelines. Flow cytometric analysis confirmed the presence of MSCs in bone marrow cultures as more than 95% of cells expressed MSC markers, CD13, CD29, CD44, CD73, and CD90 (Figure 1B,C). Less than 5% of the cells expressed CD45 and the immune activation marker, HLA-DR. The percentage of stem cell marker expression of MSCs was >90% at subsequent passages until P3 (Figure 1B,C).

### 3.2. Effect of Incubation Temperature and Period on Cell Glycosylation

**Figure 2:**
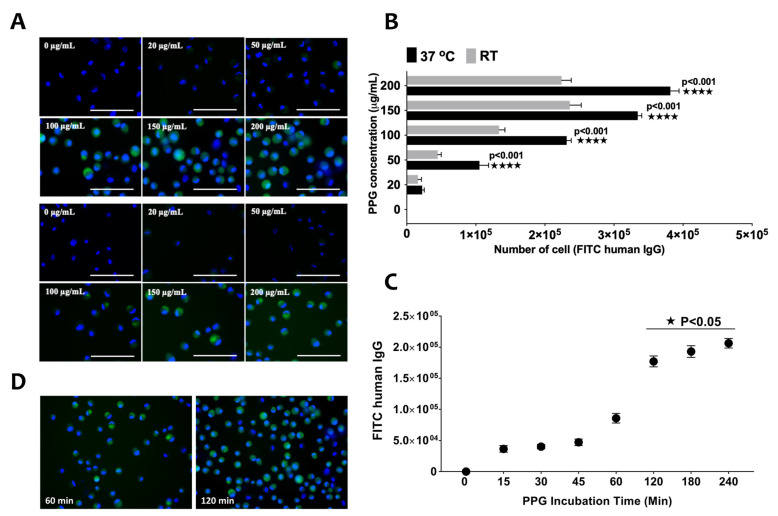
The effect of temperature and PPG concentration on the intercalation of PPG on MSCs. (**A**) The images show fluorescence staining of MSCs at each concentration of PPG. (**B**) The bar graph shows the fluorescence intensity of FITC-human IgG at 37 °C and room temperature with different PPG concentration. (**C**) The micrograph indicates the MSCs stained with FITC-human IgG at 60 and 120 min. (**D**) The graph shows the different incubation times on PPG concentrations. Scale bars indicate 100 µm (* *p* < 0.05, **** *p* < 0.001). The MSCs were counterstained with Hoechst staining (blue).

A test was conducted to determine the relationship between temperature and PPG concentration, at the optimum PPG incubation time. The conjugation of PPG to MSCs showed a concentration-dependent effect at 37 °C, whereby the conjugation could be observed at 50 µg/mL of PPG and intensified until 200 µg/mL (Figure 2A). There was less fluorescence staining in samples incubated at room temperature. Overall, the FITC-IgG (Ab) incorporation increased approximately linearly with increasing PPG concentration at 37 °C, whereby at room temperature, the incorporation was saturated at 150 µg/mL. The extent of PPG glycosylation was significantly greater at 37 °C than room temperature at PPG concentration of 50, 100, 150, and 200 µg/mL (*p* < 0.001) (Figure 2B).

Next, we tested the effect of the incubation period on the conjugation of PPG to MSCs. PPG’s conjugation to MSCs could be measured, starting from minute 15 at 37 °C (Figure 2D). There was a significant difference in PPG incorporation to the MSCs below 60 min incubation compared to 120 min and above (Figure 2C,D). The most optimum time needed for PPG incubation was 120 min. Beyond that, no statistically significant differences were seen.

### 3.3. Effect of PPG Coating on MSCs’ Proliferation and Differentiation in Monolayer Culture

**Figure 3:**
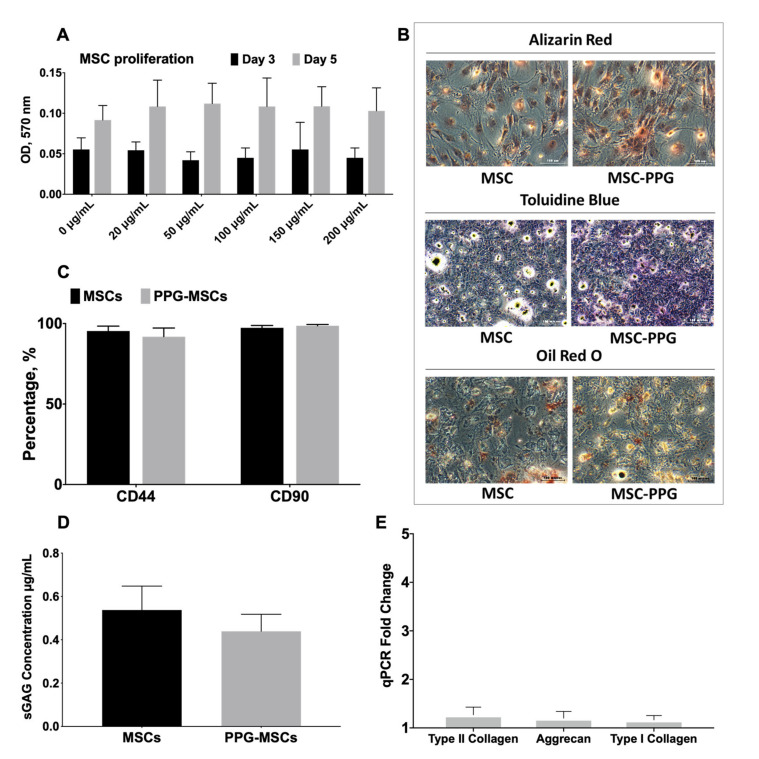
The effect of PPG coating on MSCs’ proliferation and differentiation in monolayer culture. (**A**) Bar graph showing the effect of PPG on MSCs proliferation in monolayer culture (**B**) The effect of PPG on multilineage differentiation with Alizarin red for osteocytes, Toluidine blue stain for chondrocytes and Oil Red O stain for adipocytes. (**C**) Bar graph showing the stemness marker CD44 and CD90 between MSCs and PPG-MSCs in the monolayer culture. (**D**) The effect of PPG on sGAG production in the monolayer culture. (**E**) The chondrogenic gene expression (fold change) of PPG-MSCs relative to the MSCs (control group).

Figure 3A shows the effect of PPG conjugation on MSCs on proliferation in monolayer culture. MSCs were able to proliferate despite being conjugated to PPG. No significant changes in terms of MSCs’ proliferation were observed when conjugated with PPG at concentrations 20, 50, 100, 150, and 200 µg/mL at day 3 and day 5, indicating that each PPG concentration does not affect the cell growth as compared to the control (0 µg/mL).

In terms of stemness properties, the PPG-MSCs were able to maintain their multilineage differentiation to osteocyte (Alizarin Red staining), chondrocyte (Toluidine blue staining) and adipocyte (Oil Red O staining) (Figure 3B). Figure 3C shows that PPG-MSCs equally expressed stem cell markers CD44 and CD90 as in normal MSCs. The findings indicated that PPG conjugation does not affect MSCs’ stemness.

The effect of PPG conjugation to MSCs on chondrogenic expression is shown in Figure 3D,E. No significant change was observed in terms of sGAG concentration among PPG-MSCs and MSCs (Figure 3D). Also, no substantial changes in gene expression of type II collagen, aggrecan, and type I collagen were observed between PPG-MSCs and MSCs group (Figure 3E).

### 3.4. Effect of PPG on MSCs’ Proliferation and Differentiation in 3D Culture

**Figure 4:**
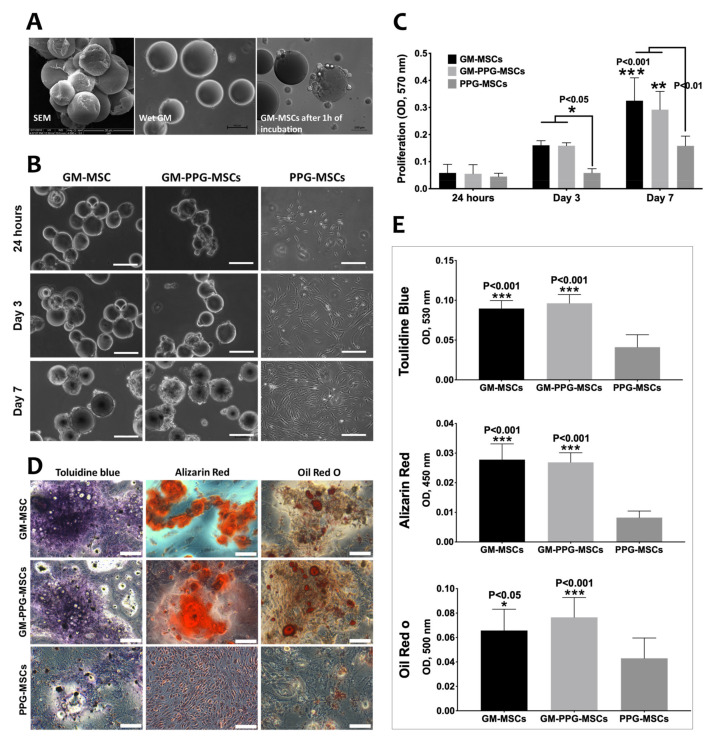
The effect of PPG on MSCs’ proliferation and differentiation in 3D culture. (**A**) Scanning electron microscopy of the GM. (**B**) The morphology of GM-MSCs as compared to the monolayer culture following day 1, 3 and 7 in culture. (**C**) The effect of PPG on MSC proliferation in a 3D culture. (**D**) The effect of antibody conjugation on multilineage differentiation of MSCs in a 3D culture. (**E**) Spectrophotometry for staining indicates that both 3D culture groups GM-MSCs and GM-PPG-MSCs groups significantly increased the staining of all lineage differentiation compared to the monolayer PPG-MSCs group. Scale bar measures 100 µm (* *p* < 0.05, ** *p* < 0.01, *** *p* < 0.001).

The GM were spherical with uniform sizes, and the surfaces were smooth (Figure 4A). They were translucent and tended to swell once immersed in water and turned transparent. The mean wet diameters of microspheres were 105.1 ± 4.6 µm. The MSCs, when cultured with GM, took as early as 1 h of incubation to attach.

The effect of PPG-MSCs’ proliferation and differentiation in a 3D culture instead of a monolayer culture was studied. The proliferation of PPG-MSCs in 3D suspension for 1, 3, and 7 days of culture was observed. Microscopic observation showed that MSCs were well attached to the GM’s surface, and there were aggregates formation among the GM (Figure 4A). During the first 24 h of culture, the number of cells among the GM-MSCs, GM-PPG-MSCs, and PPG-MSCs groups were similar (Figure 4B). On days 3 and 7, both GM-MSCs and GM-PPG-MSCs groups had significantly higher cell proliferation than monolayer PPG-MSCs groups, as expected (*p* < 0.05) (Figure 4C). However, no significant MSCs’ proliferation between GM-MSCs and GM-PPG-MSCs groups was observed from day 1 to 7 (*p* > 0.05).

Figure 4D showed a significant increase in the extracellular matrix and minerals production (demonstrated by the increased staining of Toluidine blue, Alizarin red, and Oil Red O) in both 3D culture groups (GM-MSCs and GM-PPG-MSCs) as compared to the monolayer culture (*p* < 0.05). Both GM-MSCs and GM-PPG-MSCs groups maintained their multilineage differentiation as they positively differentiated into adipocytes, osteocytes, and chondrocytes through mesodermal multilineage staining (Figure 4E). This indicated that PPG conjugation did not alter the multilineage differentiation of MSCs in monolayer and 3D culture.

**Figure 5:**
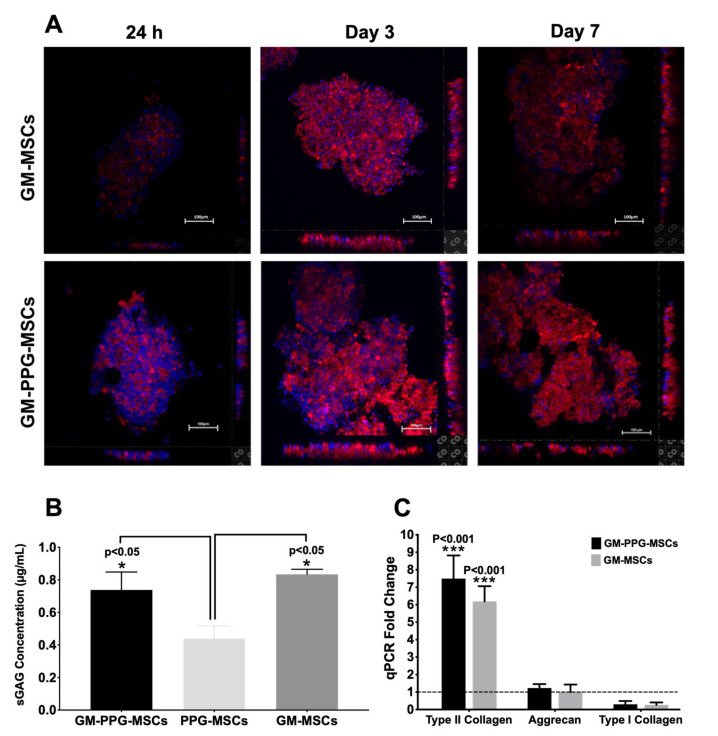
The effect of PPG on MSCs’ chondrogenic differentiation in a 3D culture. (**A**) Immunofluorescence staining of type II collagen indicates increased expression from day 1 to day 7. The bar graph shows (**B**) the sGAG production and (**C**) chondrogenic gene expression of MSCs in 3D culture. Only type II collagen gene expression is significantly increased in GM-PPG-MSCs and GM-MSCs group relative to the PPG-MSCs group (control). Scale bar measures 100 µm (* *p* < 0.05, *** *p* < 0.001).

Figure 5A shows increasing fluorescence staining of type II collagen following days of culture in the GM-MSCs and GM-PPG-MSCs groups. There was a significant increase in sGAG concentration and collagen type II gene expression in both 3D culture groups (GM-MSCs and GM-PPG-MSCs) in comparison to the monolayer PPG-MSCs group (Figure 5B,C). However, SGAG and type II collagen remained insignificant between GM-MSCs and GM-PPG-MSCs (*p* > 0.05), indicating that PPG also did not affect the chondrogenic differentiation of MSCs in 3D culture.

### 3.5. Conjugation of Antibody on PPG-MSCs in 3D Culture

**Figure 6:**
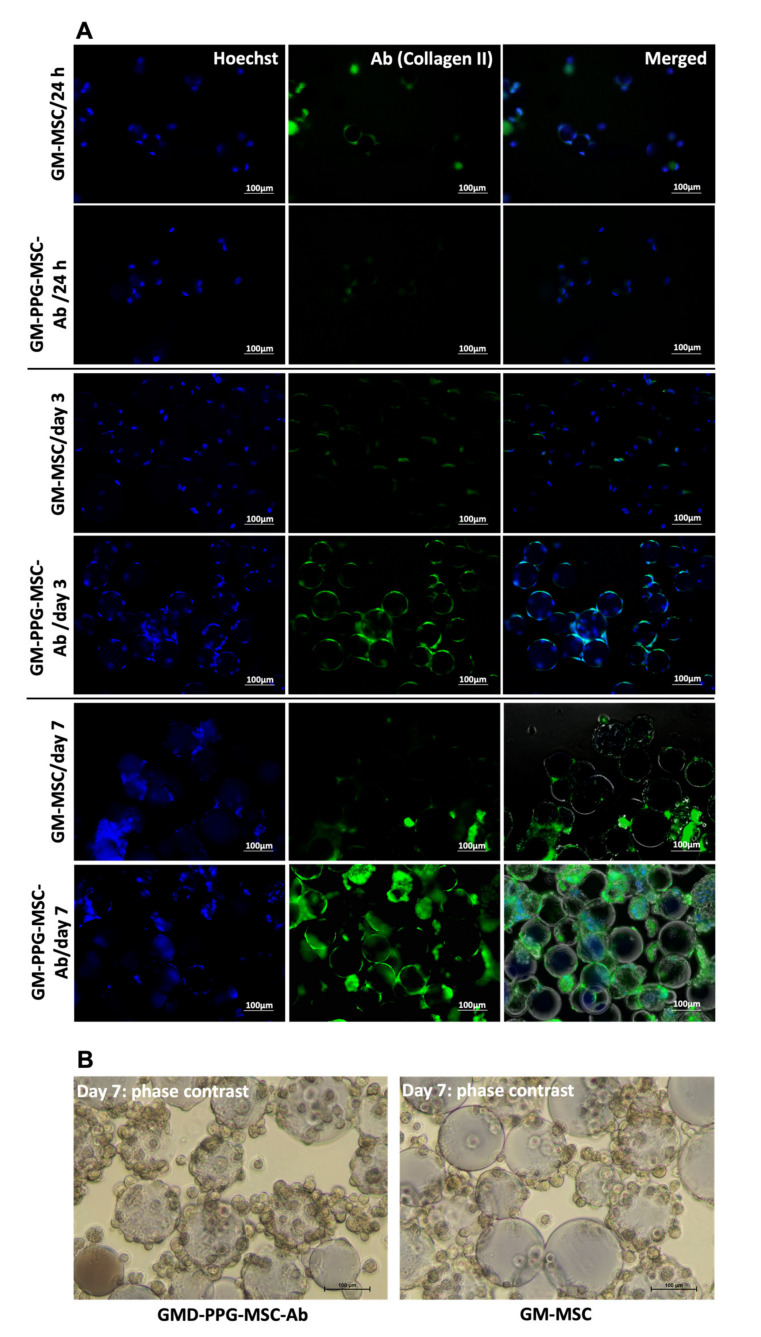
The efficiency of antibody conjugation to GM-PPG-MSCs. (**A**) Immunofluorescence staining of Hoechst dye (nucleus staining) and green fluorescent staining of type II collagen on a 3D culture. (**B**) Phase-contrast images of GM-PPG-MSCs-Ab and GM-MSCs groups that show MSCs attachment and proliferation on the surface of the GM. Scale bar measures 100 µm.

Figure 6A shows the efficiency of type II collagen antibody conjugation to the surface of PPG-MSCs. Immunofluorescence staining for anti-collagen type II was detected on day 3 and maintained until day 7, indicating effective conjugation of antibody to PPG. Antibody conjugation to PPG did not affect the MSCs as they attached and proliferated well on GM as comparable to the control group (Figure 6B).

### 3.6. Preliminary Assessment on Articular Cartilage Surface Attachment

**Figure 7:**
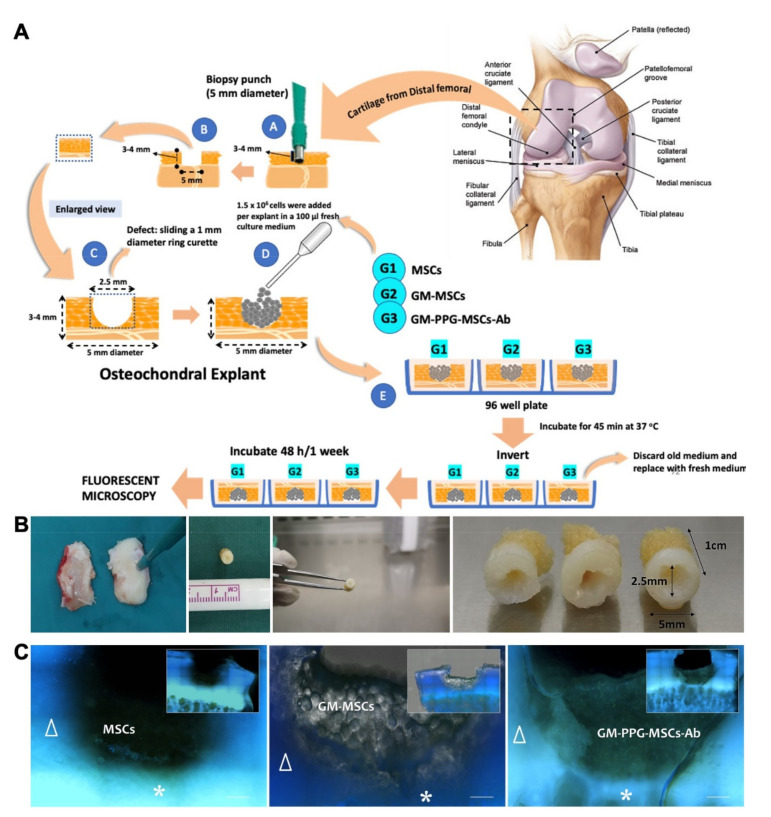
Preliminary assessment on articular cartilage explant. (**A**) Diagram shows the design flow of ex vivo implantation of MSCs, GM and GM-PPG-MSCs-Ab to osteochondral explant. (**B**) Figures showing the osteochondral explants. The explants were harvested using 5-mm punch biopsy producing a 1 × 0.25 × 0.5 cm in dimension (**C**) Immunofluorescence images of osteochondral explants incorporated with MSCs, GM-MSCs and GM-PPG-MSCs-Ab. Scale bar measure 300 µm. △—osteochondral explant, ❈—subchondral region.

After 48 h of incubation of MSCs onto the human osteochondral defect explant, GM-PPG-MSCs-Ab could be seen attaching on the osteochondral surface. Figure 7 demonstrated a clear distinction in binding capacity between the group injected with MSCs alone and GM-MSC or GM-PPG-MSCs-Ab. There is a significant increase in green fluorescence intensity in GM-PPG-MSCs-Ab compared to GM-MSCs. 

## 4. Discussion

This study highlights the effect of PPG and antibody conjugation on MSCs in monolayer and 3D culture. We demonstrated the application of glycoengineering in cell surface modification to improve targeted delivery for potential use in cartilage disease. The PPG coating did not affect MSC proliferation and differentiation in both monolayer and 3D culture. This indicates that the PPG coating is safe and effective for cell delivery.

Cellular homing is the concept of the tight integration of cells to the native tissue through intercellular adhesion [31]. There were conflicting findings in the ability of MSCs to attach to the affected tissue. In the case of MSCs delivery in OA, several studies reported the disappearance of MSCs in the joint and also at the osteoarthritic site, demonstrating their lack of integration and attachment [14,15]. To improve cell attachment, we attempted to engineer antibody conjugation to MSCs to aid the ligand binding of a specific antigen on the targeted tissue. To do this, we first conjugated protein G to the surface of MSCs, which served as the docking point for antibody conjugation to the cell through palmitation.

Palmitation or palmitoylation is a technique that describes the covalent binding of fatty acids such as palmitic acid (palmitate) to protein residues that are mainly found in cell membrane proteins [32]. Proteins can be covalently modified with various lipids; palmitic acid is an example [32]. Due to the affinity binding of protein G towards the Fc region of the antibody, protein G is an ideal candidate for mediating antibody conjugation to the cell surface [33]. Its counterpart, protein A, can also be utilized but has a lower affinity [34]. For antibody conjugation, we selected type II collagen antibody for site-specific targeted approaches, as type II collagen is the major extracellular matrix component of cartilage [35].

As the mediator for antibody–cell conjugation, the protein conjugation technique has been historically referred to as “cell painting” [36]. The intercalation of protein A or G after derivatization with palmitate to the cell surface or membrane can herald various potentials to be used for multiple applications. The protein can be fused with the Fc region of various antibodies to enhance site-specific delivery. Protein G was experimented with immunoconjugation antibodies to gold–gold sulfide nanoparticles in photothermal cancer therapy [37]. Centi and colleagues utilized protein G with gold nanorods for potential use in biosensing in nanomedicine [38]. Furthermore, in relation to the present study, previous works demonstrated that chondrocytes can be successfully coated with antibodies to chondroitin 4-sulfate and type II collagen via protein G [24].

Cell coatings with PPG are bound to be affected by multiple factors such as temperature and time. To maximize PPG coating in the culture environment, we investigated the effects of temperature in relation to the PPG concentration and incubation time. The initial result showed that PPG coating at 37 °C was better compared to that at room temperature. In addition, our results also show that the PPG coating could be detected under a fluorescence microscope starting at a concentration of 20 µg/mL at 37 °C, and became more intense at higher PPG concentrations. On the other hand, the optimum incubation time for PPG was 120 min (2 h), as suggested previously [23]. Beyond 2 h, no significant differences were noted.

Another aim of this study was to investigate whether the PPG coating is toxic and affects MSCs stemness in both monolayer and 3D culture. The results demonstrated no significant reduction/change in MSCs proliferation and stemness properties. The multilineage abilities of the MSCs were unaltered, as shown by the lineage differentiation staining of Alizarin Red for osteocytes, Toluidine blue for chondrocytes, and Oil Red O for adipocytes.

The next aim was to test the efficacy of collagen type II conjugation to the PPG-MSCs. We successfully incorporated the antibody to PPG-MSCs from day 1 to 7, as shown in Figure 6A. Further, MSC proliferation and attachment on GM with/without antibody conjugation were similar, thus suggesting the inert effect of the antibody on the cells. Previous studies also demonstrated successful antibody conjugation with PPG. As shown by Dennis and colleagues, chondroitin 4-sulfate antibody, keratan sulfate antibody, and type II collagen antibody were successfully conjugated to the auricular chondrocytes of rabbits [23]. Ko and co-workers successfully conjugated ICAM-I antibody to MSCs to enhance delivery to endothelial cells [34]. On the other hand, Lo et al. were able to incorporate 19Fc [FUT7+] to PPG on cardiosphere-derived cells and MSCs for endothelial attachment [29].

In the present study, we also compared the effect of monolayer and 3D culture environments on MSCs. Factors such as high cell density and chondrocyte phenotype maintenance are vital for ensuring successful tissue regeneration upon delivery. Chondrocytes may undergo dedifferentiation and lose their ability to produce hyaline cartilage for tissue restoration; they might instead form the undesirable fibrocartilage [39,40,41,42]. Here, we demonstrated the preservation of the MSCs ability to proliferate and differentiate chondrogenically in both monolayer and 3D culture. In addition, the present findings also confirm the increase of chondrogenic effects by MSCs in the 3D culture as seen in the previous study [25]. Microspheres, specifically gelatin microspheres, drive MSC proliferation and chondrogenic differentiation [19,20,43,44,45]. These reports indicate that microspheres might aid the maintenance of cell viability and phenotype. On the other hand, we also found that incubating the MSCs with PPG did not affect their proliferation and differentiation regardless of the cell culture environments (i.e., monolayer and 3D), demonstrating the inert effect of PPG on cell biology.

Several issues need to be addressed before we can prove that this product is suitable for use in clinical settings. We have not demonstrated the efficacy of the binding to the targeted tissues in vivo. However, our preliminary data showed that there is significant increase in binding capacity of both GM-MSCs and GM-PPG-MSC-Ab to the hyaline cartilage explants ex vivo in comparison to the MSCs alone (Figure 7). Although, it was difficult to discern a difference between the binding capacity of GM-MSCs and GM-PPG-MSCs-Ab based on the amount of GM bound to the explants in this 2D qualitative assessment, the increase in fluorescence intensity in GM-PPG-MSCs-Ab compared to GM-MSCs suggests that the GM-PPG-MSCs-Ab bound to the affected site is higher. Nevertheless, a 3D quantitative assessment and further investigations involving in vivo models are warranted in the future to prove the binding efficacy and effectiveness of the construct in cartilage regeneration.

## 5. Conclusions

We demonstrated that coating the cell surface with PPG does not interfere or affect the proliferation and stemness profile and differentiation ability of MSCs. Furthermore, culturing MSCs in a 3D culture (GM) is an efficient technique for driving MSC proliferation and differentiation compared to monolayer culture. We also successfully conjugated a collagen type II antibody to the GM-PPG-MSCs and further demonstrated in a preliminary assessment the ability of the Ab-coated MSCs to bind to an articular cartilage surface. These results suggest that GM-PPG-MSCs-Ab is a viable strategy for future targeted cell delivery.

## Figures and Tables

**Table 1:** Primer sequences used for real-time PCR experiments.

Gen Name	Forward 5′-3′	Reverse 5′-3′
Type I Col	AAGGCTTCAAGGTCCCCCTGGTG	CAGCACCAGTAGCACCATCATTTC
Type II Col	GGCAATAGCAGGTTCACGTACA	CGATAACAGTCTTGCCCCACTT
Aggrecan	ACTTCCGCTGGTCAGATGGA	TCTCGTGCCAGATCATCACC
GAPDH	GGCGATGCTGGCGCTGAGTAC	TGGTTCACACCCATGACGA

## Data Availability

Not applicable.
